# Global insights into aging: a multidisciplinary approach to understanding and addressing age-related challenges

**DOI:** 10.1093/lifemedi/lnae029

**Published:** 2024-08-20

**Authors:** Si-Yi Su, Chuting He, Jie Ren, Moshi Song

**Affiliations:** Key Laboratory of RNA Science and Engineering, CAS Key Laboratory of Genomic and Precision Medicine, Beijing Institute of Genomics, Chinese Academy of Sciences and China National Center for Bioinformation, Beijing 100101, China; University of Chinese Academy of Sciences, Beijing 100049, China; School of Future Technology, University of Chinese Academy of Sciences, Beijing 100049, China; Key Laboratory of Organ Regeneration and Reconstruction, State Key Laboratory of Membrane Biology, Institute of Zoology, Chinese Academy of Sciences, Beijing 100101, China; University of Chinese Academy of Sciences, Beijing 100049, China; Institute for Stem Cell and Regeneration, Chinese Academy of Sciences, Beijing 100101, China; Beijing Institute for Stem Cell and Regenerative Medicine, Beijing 100101, China; Key Laboratory of RNA Science and Engineering, CAS Key Laboratory of Genomic and Precision Medicine, Beijing Institute of Genomics, Chinese Academy of Sciences and China National Center for Bioinformation, Beijing 100101, China; University of Chinese Academy of Sciences, Beijing 100049, China; School of Future Technology, University of Chinese Academy of Sciences, Beijing 100049, China; Sino-Danish College, University of Chinese Academy of Sciences, Beijing 101408, China; Institute for Stem Cell and Regeneration, Chinese Academy of Sciences, Beijing 100101, China; Key Laboratory of Organ Regeneration and Reconstruction, State Key Laboratory of Membrane Biology, Institute of Zoology, Chinese Academy of Sciences, Beijing 100101, China; University of Chinese Academy of Sciences, Beijing 100049, China; Institute for Stem Cell and Regeneration, Chinese Academy of Sciences, Beijing 100101, China; Beijing Institute for Stem Cell and Regenerative Medicine, Beijing 100101, China

## Abstract

Aging has ascended to the forefront of scientific exploration, demanding a concerted global focus. The 2024 China Aging Science Conference and International Conference on Aging Biology hosted a panel discussion that brought international experts to delve into the complexities of aging research. The discussion underscores the imperative need for a multidisciplinary approach, integrating reductionist and holistic perspectives to unravel the molecular and epigenetic underpinnings of the aging process. Experts advocate for elucidating aging mechanisms and biomarkers, with a focus on translating scientific discoveries into tangible societal benefits. The discussion also emphasizes the importance of international and interdisciplinary collaborations, calling for more support to innovate for healthy aging and tackle age-related challenges.

At the 2024 China Aging Science Conference and International Conference on Aging Biology, a remarkable gathering of scientific minds took place with over 1200 attendees. The panel discussion brought together a group of international experts to delve into the complexities of aging research (Fig. 1). This collective effort by scientists around the world emphasizes the universal significance of aging as a scientific inquiry that demands global attention. The discussion underscores the need to embrace both reductionist and holistic approaches to understand and tackle the multifaceted challenges posed by aging. Topics covered included the molecular basis of aging from reductionist and holistic perspectives, the challenges and opportunities in aging research, and the importance of international and interdisciplinary collaborations in advancing aging research.

**Figure 1.**
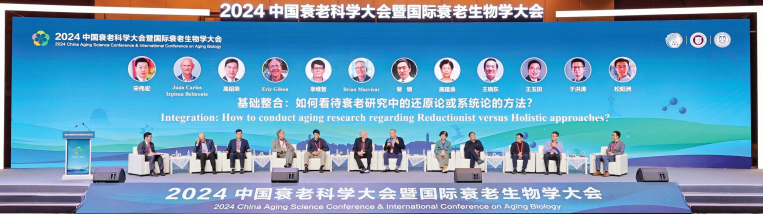
The panel discussion with international experts to explore topics related to aging research.

## Aging research through reductionist or holistic lenses

The current state of aging research is driven by the escalating global demographic shift toward an aging population. Aging, characterized by declining functions in various organs and tissues, is the primary risk factor for chronic diseases. It has been estimated that in 2023, over half of the population aged 60 and above suffer from chronic conditions, amounting to hundreds of millions of elderly individuals affected [1]. This poses a significant burden on both individuals and society. In response, advancing research in aging science, innovating and translating preventive technologies, and shifting from singular disease treatments to comprehensive aging interventions aimed at early warning and timely interventions hold strategic importance and practical value in delaying functional decline, preventing and treating age-related diseases, and promoting healthy aging.

Recognizing aging as a complex and multifaceted phenomenon, Dr. Gang Pei initiated the discussion by emphasizing the need for a balanced approach that combines detail-oriented reductionism with a holistic view. He highlighted the significance of comprehending the underlying mechanisms of aging at molecular and epigenetic levels. Dr. Pei also noted the importance of not only defining biomarkers but also deepening our understanding of the fundamental causes of aging. Furthermore, he stressed the need to bridge the gap between scientific inquiry and the general public, translating scientific advancements and theory into practical social benefits.

Dr. Juan Carlos Izpisua Belmonte echoed Dr. Pei’s perspective, agreeing with the benefits of a unified approach for advancing aging research. He acknowledged the challenge of convincing regulatory bodies about the effectiveness of geroprotective treatments, which is partly due to the complex and nuanced nature of aging. He further suggested that focusing on age-related diseases, such as those that result in cognitive decline or cardiovascular issues, could pave the way for significant progress. He also proposed that the development of medicines capable of targeting multiple aging symptoms could represent a breakthrough.

Aging is a major risk factor for chronic diseases in the elderly. Dr. Weihong Song, an expert on Alzheimer’s Disease, emphasized the urgent need to accurately and objectively assessing aging, and define the mechanisms underlying aging and related disorders. He emphasized aging study requires the coordinated effort and close collaboration from basic scientists and clinicians with multidisciplinary approaches.

As a developmental biologist, Dr. Shaorong Gao believed that cellular reprogramming was a promising avenue for cellular rejuvenation. However, as the precise expression of reprogramming factors and the duration and frequency of their induction are critical, the establishment and refinement of reprogramming standards to guide researchers in efforts to reverse cellular aging are still in demand.

Dr. Eric Gilson added that due to the profound social implications arising from the fundamental biological question that is aging, various dimensions, including social and ecological aspects, should also be integrated into the aging study. He stressed that although aging is often viewed from a social perspective, its scientific underpinnings should not be ignored. His research on coral reefs could offer unique insights into how environmental factors influence aging processes, while such interdisciplinary approaches may help researchers gain a fuller understanding of aging.

In conclusion, aging research necessitates a nuanced approach blending reductionism and holism. It is vital to comprehend aging’s molecular and epigenetic mechanisms, defining biomarkers, and bridging scientific advancements with societal benefits. While it is essential to promote close collaborations among scientists and clinicians in the field of aging research, integrating social and ecological dimensions underscores the importance of interdisciplinary approaches. Ultimately, studying aging from both social and scientific angles will lead to a deeper, more comprehensive understanding of this complex phenomenon.

## Challenges and opportunities in aging research

Regarding the challenges and opportunities, Dr. Pei highlighted two aspects. One lies in the field of research: while scientists tackle the intricate mechanisms that drive aging at the molecular level, they are also reminded that aging is a multi-tiered process influencing organ systems and the body as a whole. The other lies in the translation of basic research into societal benefits. As knowledge on aging expands, efforts could be made toward preventing or curing aging-associated diseases. This endeavor would also necessitate engagement with the public and political leaders to advocate for the importance of aging research.

Additionally, Dr. Belmonte emphasized the need for a unified approach to aging research. As aging is a complex puzzle, researchers from different knowledge backgrounds, ranging from molecular biology to epidemiology, should cooperate to deepen the understanding of genetic and environmental factors that contribute to aging.

Dr. Weizhi Ji underscored the evolutionary aspect of aging. He compared aging to the longevity of a well-crafted car, emphasizing the significance of genetic background and environmental factors. To gain a comprehensive understanding of aging, it is crucial to examine life’s early developmental stages, such as gastrulation, as these stages significantly impact our lifespan. Additionally, the interaction between genetic and environmental factors must not be overlooked.

Dr. Brian MacVicar challenged the notion of aging as a disease. With the perspective that aging, in that it is a natural progression of life, the focus should be therefore directed toward understanding the diseases that we become more susceptible to as we age. A coordinated, multidisciplinary approach that brings together genetics, epigenetics, and organ system specialists might help alleviate age-related diseases and improve the quality of life for aging populations.

As the elderly population is growing rapidly, Dr. Yunyu Shi emphasized that aging should be understood as a societal challenge. As the national demand for healthy aging has grown more pressing, researchers should aim to promote early diagnosis and intervention, as well as ultimately determine how to treat degenerative diseases.

Dr. Xiaodong Wang proposed that approaches should be validated within biological systems by employing genetic and chemical tools to delineate the stages of aging. Utilizing genetic models, including flies and nematodes, provides clear boundaries for research. He further suggested that understanding the genetic mutations that contribute to aging could be essential for the development of targeted interventions.

To facilitate the discovery of effective treatments, Dr. Yutian Wang suggested that basic and clinical researchers collaborate closely to identify true targets through repeated validation. He highlighted the challenges of the developing small-molecule drugs due to their metabolism and excretion requirements, and advocated alternative approaches, such as polypeptides, which do not require liver metabolism or kidney excretion.

Dr. Weihong Song stated that the fundamental purpose of studying aging is not simply to extend human lifespans, but more importantly to better allow individuals to live healthily with an overall higher quality of life. The world is rapidly growing older and many countries have become aged societies. As an example, nearly 300 million people in China are aged 60 or over, which accounts for 21% of the population. Given such a large proportion, aged populations are a major concern for both policymakers and wider society alike. The aging study has attracted much attention and is entering a golden age. It is crucial that we uncover the factors that trigger and influence pathological aging and determine the causes of aging-associated disorders so that we may better treat and prevent them. To further these aims, greater support from the government and society as a whole are critical.

Dr. Hongtao Yu commented that the term “aging” may not fully encapsulate the breadth of age-related processes and that a broader definition encompassing the entire life process could offer a more comprehensive scope for research. He also stressed that multidisciplinary collaboration is key to advancing the understanding and treatment of aging.

An expert in molecular biology, Dr. Zhou Songyang has always been attracted to the complex mechanisms involved in the aging process. As the aging process is an all-encompassing process, its influence ranges from one molecule to the entire body. Therefore, he concluded this topic by emphasizing the importance of multidisciplinary collaboration for dealing with the challenge of advancing aging research.

Overall, the challenges of aging research encompass its multifaceted nature, the intricate interplay of genetic and environmental factors, translational barriers, the need for extensive collaboration, and insufficient support and funding. To overcome these challenges, a comprehensive, multi-disciplinary approach is crucial, integrating basic and clinical research, fostering collaboration across fields, and engaging with the public and policymakers. Besides, increased investment and funding from governments and society are vital to support research efforts and translate findings into practical interventions that promote healthy aging.

## International and interdisciplinary collaborations

Dr. Xiaodong Wang opened the discussion by stressing the importance of encouraging young scientists to engage in aging research. He believed that senior scientists should create opportunities for young scientists to explore and innovate. He also mentioned that society should support the translation of scientific discoveries into that which has practical social value, particularly in improving health and quality of life.

Furthermore, Dr. Belmonte offered his unique insight into the distinction between academia and industry. In his opinion, academia fosters creativity and freedom in research but faces difficulties in translating knowledge into practical applications while the industry is goal-oriented but can struggle with the unpredictability of research outcomes. Hence, he appealed for an alternative model that combines the strengths of both sectors, creating an environment where scientific discoveries are robust, reproducible, and ready for clinical application.

Based on the above discussion, Dr. Zhou Songyang added that as society faces the issues brought about by aging, people look toward advancements in the scientific field for aid and relief. Researchers in academia are more familiar with the latest discoveries, while those in industry are more sensitive to the needs of society. Thus, academia and industry should seek more opportunities for collaboration, translating scientific discoveries into benefits for society.

Dr. Weihong Song described the importance of international and interdisciplinary collaboration in aging research. He highlighted that scientists need platforms for such collaboration, and therefore, they need to convince government officials of the importance of these partnerships.

Sharing a similar idea, Dr. Gilson stressed the importance of international collaboration for aging research. These collaborations allow researchers to study aging across different populations and lifestyles. By comparing aging across populations, scientists can gain insights into the basic mechanisms of aging and work toward developing effective interventions. Therefore, he noted the significance of political and financial support for international studies.

Dr. Pei believed that interdisciplinary collaboration is the first step toward further cooperation, and conferences such as the current one, where experts from different fields come together, are a great start. Initially, he proposed the integration and collaboration among researchers from diverse life science backgrounds, encouraging each participant to discover their unique contribution to aging research. Secondly, he underscored the pivotal role of social support in aging research, emphasizing the need for a collaborative effort encompassing both life science and medicine. Thirdly, he advocated for the incorporation of additional scientific disciplines, including chemistry, physics, computer science, and artificial intelligence, to foster a holistic approach to aging research. Lastly, he acknowledged the profound impact of government policies and regulations on biotechnology advancements, emphasizing the crucial role of government support. Ultimately, he advocated for aging studies to be inclusive and welcoming to all disciplines and perspectives.

In brief, international and interdisciplinary collaborations in aging research hold significant potential for advancing our understanding of the aging process and developing innovative solutions. By bringing together researchers from diverse backgrounds, these collaborations foster a rich exchange of ideas, methods, and perspectives. International collaborations enable access to diverse populations and environments, allowing researchers to study aging in different contexts and identify universal trends and factors that influence the aging process and thus facilitating the development of interventions and treatments that are more effective and widely applicable. Interdisciplinary collaborations, meanwhile, adopt a holistic approach, tackling aging-related challenges from multiple angles and facilitating the translation of discoveries into practical applications that benefit society. Still, the success of these collaborations relies on effective communication platforms and government support recognizing their importance in advancing scientific knowledge and societal impact.

## Summary

The panel discussion concluded on a high note, with a clear consensus on the importance of a multidisciplinary and collaborative approach to aging research. The gathering of international scientists not only showcased the global appeal of understanding aging but also provided a platform for shared knowledge and ideas (Table 1). The dialogue was rich with insights, emphasizing the need for precision in cellular reprogramming, the importance of considering social and ecological dimensions, and the critical role of international and interdisciplinary collaboration. The success of this meeting lies in its ability to provide direction for future research, fostering a global effort to address the challenges of aging. The commitment of the scientific community to continue this dialogue and to work together bodes well for the future of aging research and the potential to improve the quality of life for aging populations worldwide.

**Table 1.** A Summary of the panel discussion.

Topics	Highlights
Aging Research through Reductionist or Holistic Lenses	Holistic Approach to Aging: Experts advocated for a comprehensive approach to aging research, integrating detailed molecular understanding with broader social and regulatory considerations. Importance of Biomarkers and Mechanisms: Emphasis was placed on defining biomarkers and understanding the fundamental causes of aging at molecular and epigenetic levels, crucial for developing effective interventions. Potential of Geroprotective Treatments: Experts highlighted treatments targeting age-related diseases including cognitive decline, as well as suggesting targeting aging itself as potential breakthroughs in medicines to address multiple age-related diseases.
Challenges and Opportunities in Aging Research	Multi-tiered Nature of Aging: Experts stressed the importance of a holistic approach to aging, integrating molecular research with systemic impacts across multiple organ systems. Societal Impact of Aging: Aging presents significant societal challenges, requiring early diagnosis, interventions, and treatments to manage degenerative diseases and enhance the quality of life for aging populations. Interdisciplinary Collaboration and Research Scope: The significance of multidisciplinary collaboration in aging research is widely acknowledged. Experts emphasized the use of genetic and chemical tools to study aging stages and validate treatment targets. They further underscored the need to broaden the research scope to encompass the comprehensive study of aging’s its full life-cycle impact and the development of holistic treatment approaches.
International and Interdisciplinary Collaborations	Integration of Academia and Industry: Experts specifically highlighted collaboration between academia and industry to translate scientific discoveries into practical applications in aging research. They proposed an integrated model to combine academic creativity with industrial efficiency. International Collaboration for Broad Insights: Emphasis was placed on international collaboration to study aging across diverse populations, aiming to uncover universal aging mechanisms and develop globally relevant interventions.
